# Preschool children's health and its association with parental education and individual living conditions in East and West Germany

**DOI:** 10.1186/1471-2458-6-312

**Published:** 2006-12-28

**Authors:** Xianming du Prel, Ursula Krämer, Heidrun Behrendt, Johannes Ring, Hanna Oppermann, Tamara Schikowski, Ulrich Ranft

**Affiliations:** 1 Institut für umweltmedizinische Forschung (IUF) an der Heinrich-Heine-Universität Düsseldorf, Düsseldorf, Germany; 2 ZAUM – Zentrum Allergie und Umwelt, Technische Universität München and Division of Environmental Dermatology and Allergy, GSF und Technische Universität München, München, Germany; 3 Dermatologische Klinik und Poliklinik am Biederstein, Technischen Universität München, München, Germany; 4 Landesamt für Verbraucherschutz des Landes Sachsen-Anhalt, Magdeburg, Germany

## Abstract

**Background:**

Social inequalities in health exist globally and are a major public health concern. This study focus on a systematic investigation into the associations between health indicators, living conditions and parental educational level as indicator of the social status of 6-year-old children living in West and East Germany in the decade after re-unification. Explanations of observed associations between parental education and health indicators were examined.

**Methods:**

All boys and girls entering elementary school and living in predefined areas of East and West Germany were invited to participate in a series of cross-sectional surveys conducted between 1991 and 2000. Data of 28,888 German children with information on parental education were included in the analysis. Information about educational level of the parents, individual living conditions, symptoms and diagnoses of infectious diseases and allergies were taken from questionnaire. At the day of investigation, atopic eczema was diagnosed by dermatologists, blood was taken for the determination of allergen-specific immuno-globulin E, height and weight was measured and lung function tests were done in subgroups. Regression analysis was applied to investigate the associations between the health indicators and parental educational level as well as the child's living conditions. Gender, urban/rural residency and year of survey were used to control for confounding.

**Results:**

Average response was 83% in East Germany and 71% in West Germany. Strong associations between health indicators and parental education were observed. Higher educated parents reported more diagnoses and symptoms than less educated. Children of higher educated parents were also more often sensitized against grass pollen or house dust mites, but had higher birth weights, lower airway resistance and were less overweight at the age of six. Furthermore, most of the health indicators were significantly associated with one or more living conditions such as living as a single child, unfavourable indoor air, damp housing condition, maternal smoking during pregnancy or living near a busy road. The total lung capacity and the prevalence of an atopic eczema at the day of investigation were the only health indicators those did not show associations with any of the predictor variables.

**Conclusion:**

Despite large differences in living conditions and evidence that some poor health outcomes were directly associated with poor living conditions, only few indicators demonstrated poorer health in social disadvantaged children. These were in both parts of Germany increased levels of overweight, higher airway resistance and, in East Germany only, reduced height in children with lower educated parents compared to those of higher education. In both East and West Germany, higher prevalence of airway symptoms was associated with a damp housing condition, and lower birth weight, reduced height and increased airway resistance at the age of six were associated with maternal smoking during pregnancy. The latter explained to a large extent the difference in birth weight and airway resistance between the educational groups.

## Background

Social inequalities in health are a growing public health concern. Globally and historically, socio-economic status has a profound influence on health [1]. Equity in health is one of the main aims of the WHO program on health policy "Health for all" [2]. However, important social health inequalities exist all over the world and also exist in Germany. In West as well as in East Germany, mortality and morbidity increase with decreasing social status [3]. The majority of research on socio-economic status and health has focused on adults. Far less is known about the impact of socio-economic status on health during childhood. Nevertheless, what is known about socio-economic differences in children's health presents a heterogeneous picture.

On the one hand, poverty which is synonymous with very low socio-economic status is associated in industrialized countries with higher risk of death in infancy and childhood, chronic childhood diseases, many acute illness, low birth weight, obesity and mental health problems [4-8]. Poorer health in poor children is generally explained by the parents' low level of education and negative health behaviours and by the higher frequency of neonatal health problems [9]. Furthermore, exposure to environmental risks may differ in different social groups and can add another burden to groups already at higher risk of disease and may even have disparate impacts [10,11]. For instance several factors that might be expected to vary with socio-economic status have been reported to modify children's lung function, these include area of residence, outdoor air pollution, type of heating or cooking fuel used, crowding and maternal smoking during pregnancy [12].

On the other hand, atopic diseases and allergic sensitisation in both children and adults have been reported to occur more frequently in higher than in lower socio-economic groups in eastern and western industrialized countries [13-15]. Children, whose parents have a high education suffer more frequently from allergies compared to children from less educated parents, this relationship holds true in West Germany as well as in East Germany [16]. Mielck [17] reviewed 24 studies on the association between childhood asthma and socioeconomic status. He resumed that the studies did not reveal a clear picture; positive associations were as frequent as negative ones, and most studies showed no association at all. The choice of socio-economic status indicator may make a difference to the measured degree of socio-economic inequality in health [18].

After the German re-unification, tremendous social and economic changes have occurred in a few years during the 1990s in East Germany, the former socialist German Democratic Republic. This might serve as a time-lapse picture for future developments in other Eastern European countries of transient societies. We have already described the increase in social disparities with respect to health related living conditions of 6 year old children within East Germany between 1991 and 2000 [19,20]. This increase was especially true for "living near busy roads" and "maternal smoking".

In this study, we focus on a systematic investigation into the associations between health indicators, living conditions and parental educational level as indicator of the social status of 6-year-old children living in West and East Germany in the decade after re-unification. Beside questionnaire based information, we also used "objectively" measured variables to define health indicators. As parental education can not directly be causally related to the children's health we also sought after explanations of observed associations between parental education and health indicators. Observational studies on the relationship between the prevalence and trends of childhood diseases, health related living conditions and parental education comparing East and West Germany are not available so far.

## Methods

### Study population

The study data was collected within a large study in East and West Germany investigating the health effects in school beginners (6-year-old) of the changing environmental and socio-economic conditions after re-unification between 1991 and 2000. Details of the study design were presented elsewhere [21-23]. In brief, all boys and girls living in geographically pre-defined areas of East and West Germany and entering the elementary school between 1991 and 2000 in East and in 1991, 1994, 1997 and 2000 in West Germany were eligible to participate. Rural areas without heavy industrial impact were located in Salzwedel, Gardelegen, Osterburg and Kloetze in East Germany and in Borken and Borken District in West Germany. Urban areas with industrial impact and strong traffic burden were selected in Halle, Leipzig and Magdeburg in East Germany and in Duisburg, Essen and Cologne in West Germany. The financial support of the study and the participation potential of the local health authorities were not constant over the whole study duration. Therefore, different numbers of pre-defined areas determined the entire investigation area in each year and, in consequence, led to different sample sizes. A letter was mailed to the parents inviting the child to participate in the study and to complete a standardised questionnaire. On the day of investigation, the questionnaire was checked by a physician and any missing answers subsequently completed by the parents. In the investigation years 1991, 1994, 1997 and 2000 and in pre-selected parts of the study areas, the children were asked to undergo a dermatological examination and to provide a blood sample. Additionally, with the exception of the areas in East Germany in the year 2000, every second child was invited to have its lung function tested. The overall response rate to the questionnaire was 83% and 71% in East and West Germany, respectively. In those children eligible, the response rates for participation in the dermatological examination, donation of a blood sample and testing the lung function were 84%, 73% and 73%, respectively, in East Germany and 88%, 69% and 69% in West Germany. Written informed consent of the parents of the participating children was obtained. The ethical committee of the Medical Association of Saxony-Anhalt approved the study.

### Parental education

Information about school education of the parents was taken from the questionnaire., A suitable measure of the parental educational level was chosen by classifying the school education into the two categories of no more than 10 years school and more than 10 years school. The parent or partner with the highest school grade defined the parental educational level of the child. To avoid ambiguity in classification of the parental education, we only included children whose parents were of German nationality. This exclusion resulted in an average loss of 8% in the industrialized areas of East and of 26% of West Germany, and of 1% in the rural areas of East and 3% of West Germany. Obviously, the use of a dichotomous variable for the educational level does not sufficiently account for the very low educated parents whose children were the most disadvantaged in many aspects, e.g. the living conditions [19,20]. However, a further subdivision of the lower educational level into the two categories of "less than 10 years school" and "10 years school" result in a classification which differs in interpretation between the two parts of Germany considering their different schooling systems before re-unification. In the former German Democratic Republic, ten years of schooling was compulsory. Consequently in East Germany, only six per cent of the participating children had parents with less than 10 years school.

### Individual living conditions

In the questionnaire, the parents were asked to provide information on the child's living conditions. Altogether, six individual living conditions, which were considered relevant for the child's health, were investigated: The question about the number of sisters or brothers of the child resulted in the family condition "single child". The per capita living space of the child's home could be defined by using the two questions "How many people are living in the child's home?" and "How many square meters is the child's home?". The living space was considered small if it was below 20 m^2 ^per person ("small living space"). If an oven heated with fossil fuel or cooking with gas existed then "unfavourable indoor air" in the child's home was assumed. Affirmation of the question "Would you characterise your home as damp?" indicated a "damp housing condition". "Maternal smoking during pregnancy" was recorded. It is often highly correlated with current maternal smoking and as such can be considered as an indication of the child's exposure to environmental tobacco smoke throughout life. Traffic exposure of the children was determined by the distance of the children's home to the nearest busy road, and "living near a busy road" was stated if the distance was less than 50 m.

### Health indicators

Altogether, 14 variables indicating the child's health were investigated in this study. Of these, six health indicators representing a range of infectious and atopic diseases and symptoms were selected from the questionnaire data, namely "bronchitis ever diagnosed by a physician", "more than 4 colds during the last 12 months", "frequent cough without cold", "sneeze attacks during the last 12 months", "allergy ever diagnosed by a physician" and "eczema ever diagnosed by physician". Information regarding "birth weight" of the child was also provided by questionnaire. Seven further indicators were determined independent from the questionnaire: On the day of investigation, a physician examined the child's skin and decided on the diagnosis of an atopic eczema ("atopic eczema on the day of investigation"). Across all surveys, a standardized diagnostic procedure (SCORAD) [24] was used, and one of the authors (JR) has supervised the training of the physicians. Atopic sensitisation was tested by determining specific immuno-globulin-E (IgE) antibodies against common allergens in the blood serum of the child by an enzyme immuno assay (Radio Allergo Sorbent Test (RAST), Pharmacia & Upjohn, Uppsala, Sweden). Based on the results of the RAST, participants were classified as having "specific IgE grass pollen positive" or "specific IgE house dust mite positive" if the specific IgE concentrations against grass pollen and house dust mite, respectively, were greater than 0.35 kU/l. Height and weight were measured on the day of examination and "body height" used as a general measure of physical development. A body mass index (BMI = body weight/(body height)^2 ^[kg/m^2^]) greater than 19 kg/m^2 ^provided an indication of "overweight" Using a body plethysmograph for a sub-group of the participants, "airway resistance" and "total lung capacity" were available as indicators of lung function. Details of the measurement procedure were presented elsewhere [23]. An increased airway resistance indicates an obstruction of the airways. For children, higher total lung capacity is related to better lung development. To correct for height, gender and age, total lung capacity was provided as percent predicted. A group of healthy children aged 5 to 10 years and living in the West German study areas in 1991 was used as standard for prediction.

### Statistical analysis

As the changes of living and environmental conditions took very different courses in East and West Germany after re-unification and the educational systems in East and West Germany featured many differences before re-unification a separate statistical analysis of the study data was done for the two parts of Germany.

The statistical significance of the difference between two groups was tested using t-test and chi-square test for continuous and dichotomous variables, respectively. A linear time trend across the surveys was checked using the Cochran-Armitage test for dichotomous variables, and for continuous variables, a significance of linear regression on the year of survey was determined using the t-test.

A two-step process of multiple regression analysis was applied to reveal, in the first step, associations between parental education and health indicators and to examine, in the second step, the additional explanatory potency of the living conditions. As health indicators were the dependent variables parental educational level was the independent variable in the first step regression model which also included the three co-variables year of investigation, residency and gender as potential confounders. For the second step, the regression model from the first step was expanded by adding the six living conditions as further independent variables. For dichotomous outcome variables, we used logistic regression, and the linear multiple regression approach was used for continuous outcome variables. Interaction terms between year of investigation and parental educational level were incorporated, and the results of a stratified analysis are presented, if the interaction was statistically significant (p < 0.05). Only children with complete information regarding all dependent and independent variables were included in the respective regression model. The regression results with respect to the associations of the health indicators with educational level and the six living conditions were presented in the case of logistic regression as adjusted odds ratios and in the case of linear regression as adjusted standardized means differences, both together with 95% confidence intervals. The adjusted standardized means difference (MD) was calculated as the ratio of the expected adjusted change of the dependent variable Δy by a unit-increase of the respective independent variable and the sample mean y¯
 MathType@MTEF@5@5@+=feaafiart1ev1aaatCvAUfKttLearuWrP9MDH5MBPbIqV92AaeXatLxBI9gBaebbnrfifHhDYfgasaacH8akY=wiFfYdH8Gipec8Eeeu0xXdbba9frFj0=OqFfea0dXdd9vqai=hGuQ8kuc9pgc9s8qqaq=dirpe0xb9q8qiLsFr0=vr0=vr0dc8meaabaqaciaacaGaaeqabaqabeGadaaakeaacuqG5bqEgaqeaaaa@2E3D@ plus unity: MD = 1 + Δy/y¯
 MathType@MTEF@5@5@+=feaafiart1ev1aaatCvAUfKttLearuWrP9MDH5MBPbIqV92AaeXatLxBI9gBaebbnrfifHhDYfgasaacH8akY=wiFfYdH8Gipec8Eeeu0xXdbba9frFj0=OqFfea0dXdd9vqai=hGuQ8kuc9pgc9s8qqaq=dirpe0xb9q8qiLsFr0=vr0=vr0dc8meaabaqaciaacaGaaeqabaqabeGadaaakeaacuqG5bqEgaqeaaaa@2E3D@.

All calculations of the statistical analysis were performed using the SAS statistical software package, Version 9.01 (SAS Institute, Cary, NC).

## Results

Information on parental education was available for 28,888 children with German nationality, comprising 90.4% of all participating children. In table 1, the study size in terms of years of investigation, urban/rural residency and East or West Germany is presented in more detail. The median age of the children was 6.3 years with a range from 5.6 to 7.1 years, and the proportion of girls was 49.2%.

### Distributions of health indicators, living conditions and parental education

The distributions of health indicators, living conditions and parental education are described in terms of prevalence, mean and standard deviation, respectively, for East (table 2) and West Germany (table 3) separately. The prevalence of parents with lower school education was 65.2% in West Germany and 51.9% in East Germany. Tables 2 and 3 clearly demonstrate the large prevalence differences of the living conditions between East and West Germany. Unfavourable indoor conditions with respect to heating and cooking and dampness were more frequently reported in the East than in the West, but with respect to environmental tobacco smoke exposure as assessed by maternal smoking during pregnancy, the conditions were worse in West Germany. A single child was more common in East than in West Germany. Living space was smaller in East Germany. Differences in health indicators between East and West were much less apparent than in living conditions.

Furthermore in tables 2 and 3, the influence of urban/rural residency and year of investigation on the study variables was documented. In general, living conditions were more favourable at a rural than an urban residence in both parts of Germany. Health indicators with the exception of sensitisation and lung function were also better in rural than urban areas. For all living conditions in East Germany, we observed highly significant time trends: The number of single children and those living near a busy road increased over time, the latter peaking in the mid-nineties. Furthermore, the frequencies of small living place, unfavourable indoor air and damp housing condition decreased over time, the latter also with a peak in the mid-nineties. This was already described and discussed in more detail elsewhere [19]. Time trends in health indicators were heterogeneous: The prevalence of frequent cough and eczema at the day of examination decreased, whereas sneeze attacks, ever diagnosed eczema, overweight, height, birth weight as well as total lung capacity increased. In West Germany, changes over time in regards to living conditions and health variables were present, but to a lesser degree compared to East Germany (table 3).

With the exception of "unfavourable indoor air" in West Germany, all living conditions were significantly (p < 0.1) associated with parental educational level (table 4): Less educated parents more frequently had a single child, lived in a small or damp living place or near a busy road, or heated with coal or gas. The higher prevalence of maternal smoking in families with lower education of the parents was most striking. This also was described and discussed in more detail elsewhere [20].

In table 4, the crude associations between health indicators and the parental education are presented too. Parents with higher educational level more frequently reported symptoms and diseases than parents with lower level; the only exception was frequent cough. Also sensitisation against grass pollen and house dust mite was more frequently observed among children with parents of higher education. Overweight, small height and low birth weight however were significantly more frequent in children of less educated parents in both parts of Germany, also elevated airway resistance, but significantly in West Germany only.

### Results of regression analyses

The focus of this study was on the association between educational level and children's health and, further on, to find out whether this association was changed taking into account living conditions as predictors of the health indicators. Therefore, we applied the two-step statistical regression modelling. The results of this regression analysis are presented in tables 5 and 6 separately for East and West Germany. The crude associations of the parental education with the children's health, as presented in table 4, were essentially confirmed by the odds ratios and standardized means differences adjusted for gender, residency and year of investigation at the first step of the regression analysis (tables 5 and 6). In the second step, the regression model of the first step was augmented by the six living conditions as independent variables. The independent associations between the living conditions and health indicators are also shown in tables 5 and 6. Most of the health indicators were influenced by living conditions: Single children had more allergies and symptoms of allergies and were more often sensitised (significantly only against grass pollen in West Germany). They also feature more often overweight, were taller and their birth weights were lower. For unfavourable indoor air in East Germany, the frequency of colds was increased, but of eczema and sensitisation decreased. A damp home showed an unfavourable health impact signified by most of the health indicators. Maternal smoking during pregnancy was associated with lower birth weight and current height, increased airway resistance and frequent cough, but, especially in East Germany, with decreased frequency of atopic symptoms. Traffic exposure, indicated by a home address near a busy road, consistently had a negative impact on the children's health.

Comparing the adjusted measures of the association between parental education and health indicators of the second step with the respective measures of the first step in tables 5 and 6, one can assess the extent by which the living conditions explained the educational influences on the child's health. We could roughly identify four different patterns:

(i) Influence of parental education but no change after adjusting for living conditions: Higher educated parents reported diseases ever diagnosed by a physician and number of colds significantly more frequent than less educated parents. The odds ratios describing these associations did not change or only marginally change when adjusting for living conditions. The results were very similar in East and in West Germany, but with the one exception in East Germany that the association between parental education and ever diagnosed allergy was stronger in 1991 than in 2000. This is mainly due to more reports of higher educated parents in 1991 than in the years after.

(ii) Marginal influence of parental education with change after adjusting for living conditions: The odds ratio for the association of frequent cough and sneezing with lower parental education was reduced after inclusion of living condition factors mainly due to the strong influence of damp housing conditions. After adjusting, the odds ratio indicates more positive reports in the higher educated group. These results were also very similar in both East and West Germany.

(iii) Influence of parental education and explanation by living conditions: Overweight was more frequently observed for children with less educated parents than with higher educated. This association did not change after adjusting for the influential factor "single child" in East Germany, but in West Germany. The association of positive specific IgE sensitisation with high parental educational level in East Germany could partly be explained by living condition factors. This is mainly due to the association with "unfavourable indoor air". The lower birth weight of children from less educated families could be explained by the two factors "single child" and "small living space" and the higher prevalence of maternal smoking during pregnancy in the group with less educational level. This latter influence was stronger in West Germany and in East Germany in the year 2000 than in East Germany 1991. The differences in mean of airway resistance between the educational groups also were reduced after inclusion of lifestyle factors. This also was mainly due to maternal smoking, and again this effect was stronger in West Germany than in East Germany 1991.

(iv) No association with parental education: This was true for the total lung capacity (% predicted) and an atopic eczema at the day of investigation.

## Discussion

In this study we observed strong associations between parental education and health indicators of children. Higher educated parents reported more diagnoses and symptoms than less educated. Children of higher educated parents were also more often sensitised against grass pollen or house dust mites, but had higher birth weights, lower airway resistance and were less overweight at the age of six. Furthermore, we saw most of the health indicators significantly influenced by one or more living conditions. Particularly, a damp housing condition was significantly and consistently associated with increased prevalence of airway symptoms in both parts of Germany, and, especially in East Germany, children living near a busy road showed airway symptoms more frequently. The total lung capacity and the prevalence of an atopic eczema at the day of investigation were the only health indicators which did not show associations with any of the predictor variables.

As mentioned in the introduction, health indicators denoting infectious diseases (bronchitis, more than 4 colds in the last 12 months, frequent cough, airway resistance) or foetal and infant development (birth weight, height, total lung capacity) were expected to be negatively associated with educational level as indicator of social status, e.g. we would observe a higher prevalence of bronchitis ever diagnosed with the lower social status. Indications of atopic diseases and sensitisations, however, are expected to occur more frequently in the higher social group. Our results differ in several indicators from these expectations. We considered different mechanisms driving the associations between parental education and health indicators:

### Reporting bias

As in many of the population-based studies examining the relationship of socio-economic status to childhood disease [25], we used parental reporting to assess the respiratory outcomes. A combined analysis of questionnaire based information from studies done in children from Eastern and Western Europe and North America also revealed that diagnoses of allergies were more frequent reported in groups with higher educated parents and this was true for Eastern and Western countries [15]. Differences in reporting behaviour among parents of different social classes may bias such associations. Under-reporting or reduced access to healthcare might explain a decreased prevalence of the diagnoses with low parental education. Furthermore, the higher prevalence of bronchitis, allergy and eczema in children with high parental education could also be explained in part by differences in the perception of severity of diseases. Highly educated parents might seek medical advice in mild cases of bronchitis whereas parents with a low level of education seek medical advice in more severe cases only [15]. Therefore, part of the difference in diagnoses and symptoms of children with differently educated parents found in our study might be due to over-reporting of higher educated parents or under-reporting of less educated parents. However, the extent of this bias is difficult to assess. A comparison between "eczema at the day of investigation" which was determined independently from reporting and "eczema ever diagnosed" was already done with data from this study elsewhere [26] and demonstrated that under-reporting of less educated parents was most likely. Higher educated parents from East Germany reported allergy ever diagnosed by a physician more frequent in 1991 than in 2000 which was not true for less educated parents. This may hint to over-reporting of higher educated parents shortly after the re-unification when an allergy diagnosis perhaps became "fashionable".

### Living conditions as explanatory factors

Taking the individual living conditions of the children into account and using a two-step regression analysis approach, we looked for possible explanations of the observed health indicator differences between the two parental educational levels. None of the living conditions included in our evaluation could explain the social differences in reporting of doctor-diagnosed diseases. This does not necessarily mean that these differences are completely due to over-reporting and do not exist. Several studies conducted in different European countries already reported higher prevalence of eczema, hay fever and sensitisation to inhalant allergens among children, adolescents and adults of the most advantaged socio-economic group [13,15,27,28]. Factors related to "hygiene" are known to modify factors for allergic diseases and might be differently distributed between social groups. For instance, living as a single child in a family is related to "hygiene" (less infection in early age) and is a strong known determinant of allergic disease and sensitisation. This latter could again be demonstrated in our study. However, in this study a single child was more frequently observed in families with low educated parents. Therefore this factor could not explain lower prevalence of allergies and sensitisations in low educated families. Other factors not measured such as day care might be responsible for these differences. The following lifestyle factors, however, were found to account for differences in health indicators according to level of parental education.

To live as a single child in a family was not only a risk factor for allergies, but also a risk factor for overweight in both parts of Germany. In West Germany, this partly explained the higher prevalence of overweight in children with less educated parents. Living in damp housing conditions was a risk factor for sneezing and frequent cough and partly explained the prevalence of these symptoms in families with lower educational background because this condition was more frequent in these families. After adjusting for these conditions the odds ratio for educational differences demonstrated higher reporting in higher educated families which might again be due to over-reporting or due to the fact that these symptoms could as well be symptoms of allergic disease. Similar results were gained from the combined analysis of questionnaire based information from studies done in children from Eastern and Western Europe and North America [15]. Here, nocturnal cough and wheezing was more prevalent in groups with low educated parents in Eastern countries and more prevalent in groups with highly educated parents in Western countries where these symptoms were related to allergic diseases. Unfavourable indoor air by heating with fossil fuels or cooking with gas which was more prevalent in less educated families in East Germany was observed as a protective factor against sensitisation with pollen and partly explained differences in pollen sensitisation between groups of different educational level. A protective effect of indoor heating with fossil fuels was already demonstrated for Bavarian families [29], but remained largely unexplained. Maternal smoking during pregnancy was more prevalent in groups with lower parental education. Maternal smoking during pregnancy was also the strongest identified risk factor for low birth weight of the child. This factor mainly explains the lower birth weight of children from less educated parents. Interestingly, this factor only became relevant in East Germany for children born after the re-unification (significant interaction between year of investigation and educational level). This is due to the fact that the prevalence of "smoking during pregnancy" increased in East Germany in the less educated groups in contrast to other groups [20]. It is a well-known fact that smoking during pregnancy leads to a low birth weight of the child [30]. Maternal smoking also partly explained the higher mean airway resistance of children from less educated parents.

### Limitations

Several limitations of this study must be considered. First, the series of cross-sectional surveys were designed to investigate air pollution health effects. Data on socio-economic status, indoor air conditions and other individual living conditions of the children were collected to control for potential confounding. Therefore, only the small collection of six living conditions was available for this study. Likewise, the assessment of the socio-economic status was restricted to the parental educational level. Second, environmental conditions as air pollution dramatically changed during the ten-year observation period in East Germany. Using the year of investigation to control for these changes in the regression modelling assumes a linear time course which does not sufficiently represent the true non-linear time course and, therefore, might yield residual confounding. Third, sensitisation tests, dermatological examination at the day of investigation and lung function testing had to be restricted to parts of the whole study group causing a reduction of 10 to 20 per cent in the total study group (table 1). Therefore, the question of a selection bias arises. We compared the distributions of parental educational level between the three sub samples and their respectively remaining samples. The prevalence of children with the same parental educational level differed less than 3 per cent between the respective sub samples. Furthermore, we repeated the regression analyses of the health indicators based on questionnaire now using the three sub samples. Taking into account the reduced power due to the smaller samples sizes, the results were consistent with those presented in tables 5 and 6 (details not shown). Fourth, we used a dichotomous variable for classifying parental education to account for the different schooling systems in East and West Germany. As a consequence of that, differing associations for the very low educated subgroup could have been overlooked in the regression analysis. Therefore in a supplemental regression analysis for the outcomes assessed by questionnaire, we used two binary dummy variables for a three-level classification scheme of the parental education (less than, equal or more than 10 years school). Though the adjusted odds ratios of the lowest versus the highest educational level generally were slightly more divergent from the null value than the odds ratios of the middle versus the highest level, the results were consistent with those presented in tables 5 and 6 (details not shown).

## Conclusion

Most of the health indicators were significantly associated with parental education and one or more living conditions, the exceptions being total lung capacity and prevalence of an atopic eczema at the day of investigation. The seemingly protective influence of low educational level on reported diagnoses and airway symptoms was probably attributable to reporting bias at least in part. The prevalence of sensitisation against grass pollen and house dust mite was lower in children with low educated parents, but this remained unexplained. Despite large differences in living conditions and evidence that some poor health outcomes were directly associated with poor living conditions, only few indicators demonstrated poorer health in social disadvantaged children. These were in both parts of Germany increased levels of overweight, higher airway resistance and, in East Germany only, reduced height in children with lower educated parents compared to those of higher education. In both East and West Germany, higher prevalence of airway symptoms was associated with a damp housing condition, and lower birth weight, reduced height and airway resistance at the age of six were associated with maternal smoking during pregnancy. The latter explained to a large extent the difference in birth weight and airway resistance between the educational groups.

## Competing interests

The author(s) declare that they have no competing interests.

## Authors' contributions

UR proposed the idea for the article. XDP wrote the first draft, supervised by UR. HO coordinated the study in East Germany. HB and JR were responsible for the evaluation of allergy related variables. UK was main investigator of the study in East and West Germany and together with UR edited subsequent drafts. TS commented on the draft. All authors reviewed the final version of the manuscript.

## Pre-publication history

The pre-publication history for this paper can be accessed here:



## Figures and Tables

**Table 1:** Study population of 6-year-old children in East and West Germany by year of investigation and urban/rural residency. Number of participants of German nationality with complete information on parental education and portions of these participants with investigation of sensitisation (RAST), atopic eczema or lung function test.

***Year***	***Sample size (percentage with RAST, dermatological investigation and lung function test)***				
	
	***East Germany***		***West Germany***		***Total***
	***Urban* ^***a***^**	***Rural* ^***b***^**	***Urban* ^***c***^**	***Rural* ^***d***^**	
			
***1991***	3108 (32.4; 9.1; 24.3)	916 (40.9; 0.0; 25.7)	2554 (16.9; 20.9; 5.4)	359 (58.5; 68.8; 20.1)	6937 (29.2; 15.3; 17.2)
***1992***	1225	924	--^e^	--^e^	2149
***1993***	973	873	--^e^	--^e^	1846
***1994***	3331 (24.6; 17.2; 8.5)	890 (33.3; 31.7; 26.7)	396 (70.5; 37.1; 45.5)	265 (79.6; 87.7; 44.2)	4882 (32.9; 25.2; 16.8)
***1995***	1061	714	--^e^	--^e^	1775
***1996***	965	667	--^e^	--^e^	1632
***1997***	2095 (16.9; 16.5; 10.5)	561 (36.4; 47.1; 28.9)	356 (64.0; 82.9; 31.5)	429 (53.1; 58.3; 11.0)	3441 (28.1; 33.6; 15.7)
***1998***	862	321	--^e^	--^e^	1183
***1999***	826	356	--^e^	--^e^	1182
***2000***	1662 (17.0; 16.2; 0.0)	336 (29.5;38.4; 0.0)	1540 (12.3; 15.8; 13.1)	323 (41.2; 52.9; 44.6)	3861 (18.2; 21.1; 8.9)
***Total***	16108 (15.3; 9.1; 7.8)	6558 (14.9; 10.3; 9.5)	4846 (22.2; 25.2; 13.0)	1376 (56.8; 65.3; 27.6)	28888 (18.3; 14.8; 10.0)

^a ^Leipzig, Halle and Magdeburg; ^b ^Salzwedel, Gardelegen, Osterburg and Kloetze; ^c ^Duisburg, Essen, and Cologne; ^d ^Borken; ^e ^no investigation

**Table 2:** Distribution of health indicators, living conditions and parental educational level of 6-year-old children in East Germany stratified by urban/rural residency and year of investigation.

	**Mean ± standard deviation/Prevalence [%]**														
	
	***Total***	***Residency***^**a,b**^			**Year of investigation**^***c,d***^										
		***Urban***^***e***^	***Rural***^***f***^		***1991***	***1992***	***1993***	***1994***	***1995***	***1996***	***1997***	***1998***	***1999***	***2000***	
***Health indicators***															
			
Airway resistance [10^-1^•kPa•s/l]	6.67 ± 1.64	6.64 ± 1.59	6.71 ± 1.73	-	6.76 ± 1.74	--^g^	--^g^	6.52 ± 1.56	--^g^	--^g^	6.63 ± 1.44	--^g^	--^g^	--^g^	-
Total lung capacity [% predicted]	104 ± 11	102 ± 10	105.5 ± 11	*	101 ± 10	--^g^	--^g^	106 ± 10	--^g^	--^g^	107 ± 11	--^g^	--^g^	--^g^	*
Birth weight [10•g]	333 ± 54	331 ± 54	336 ± 54	*	327 ± 51	331 ± 59	333 ± 53	332 ± 54	334 ± 56	335 ± 52	332 ± 53	336 ± 52	336 ± 56	340 ± 55	*
Body height [cm]	120.1 ± 5.5	119.9 ± 5.5	120.6 ± 5.5	*	119.5 ± 5.4	119.7 ± 5.4	120.0 ± 5.8	120.5 ± 5.4	120.0 ± 5.6	120.5 ± 5.3	120.4 ± 5.6	120.3 ± 5.7	120.4 ± 5.8	120.0 ± 5.5	*
Overweight, BMI > 19 kg/m^2^	5.6	5.5	5.8	-	3.5	3.9	5.2	5.7	5.8	6.1	7.0	7.6	6.3	8.0	*
Bronchitis, ever diagnosed	48.1	49.7	44.3	*	56.0	50.3	47.2	50.6	44.9	45.0	44.9	43.6	44.0	39.4	*
More than 4 colds in last 12 months	13.1	14.7	9.1	*	16.1	14.2	11.6	13.4	9.8	11.1	11.7	12.0	14.6	12.7	*
Frequent cough	6.4	7.3	4.2	*	10.0	7.4	6.2	6.0	6.4	4.7	4.9	4.5	5.3	4.5	*
Sneeze attacks in the last 12 months	5.0	5.7	3.2	*	4.8	2.9	3.0	4.4	4.0	4.2	6.7	6.6	6.5	7.7	*
Allergy, ever diagnosed	9.3	9.6	8.5	*	11.5	8.4	9.2	7.7	7.4	8.9	9.0	9.7	9.4	10.8	-
Eczema, ever diagnosed	16.9	16.1	15.4	-	14.7	12.1	13.8	15.0	15.0	16.4	18.0	18.1	20.9	20.0	*
Atopic eczema, day of investigation	11.7	12.4	10.1	-	17.7	--^g^	--^g^	12.5	--^g^	--^g^	11.2	--^g^	--^g^	6.5	*
Specific IgE grass pollen positive	11.3	10.9	12.2	-	11.7	--^g^	--^g^	10.8	--^g^	--^g^	10.5	--^g^	--^g^	12.1	-
Specific IgE house dust mite positive	9.8	9.6	10.4	-	10.1	--^g^	--^g^	9.2	--^g^	--^g^	9.7	--^g^	--^g^	10.8	-
***Living conditions***															
															
Single child	38.2	41.5	30.3	*	30.9	30.2	33.6	38.5	39.5	41.7	46.6	45.4	41.2	44.7	*
Small living space	46.3	48.1	41.8	*	58.7	49.3	46.2	53.4	42.6	38.6	43.2	36.8	33.2	32.1	*
Unfavourable indoor air	50.8	53.8	43.5	*	62.5	73.7	61.5	50.6	58.7	47.3	37.1	36.6	31.7	27.2	*
Damp housing condition	10.5	11.7	7.6	*	9.4	11.0	11.4	11.6	13.3	13.5	10.2	9.8	9.1	6.0	*
Maternal smoking during pregnancy	9.2	10.1	7.3	*	12.2	7.6	7.5	6.3	7.0	6.4	10.7	12.1	11.5	12.4	*
Living near a busy road	59.9	61.8	55.2	*	55.6	59.8	62.8	63.0	63.3	60.8	59.6	58.8	58.6	57.1	-
***Parental education***															
															
Low parental educational level	51.9	49.9	56.7	*	48.6	52.4	50.1	50.2	50.9	49.7	53.8	58.0	57.1	54.7	*

^a ^t-test (continuous variables); ^b ^chi-square test (dichotomous variables); ^c ^t-test for correlation (continuous variables); ^d ^trend test (dichotomous variables); ^e ^Leipzig, Halle and Magdeburg; ^f ^Salzwedel, Gardelegen, Osterburg and Kloetze; ^g ^not investigated; * p < 0.05

**Table 3:** Distribution of health indicators, living conditions and parental educational level of 6-year-old children in West Germany stratified by urban/rural residency and year of investigation.

	**Mean ± standard deviation/Prevalence [%]**								
	
	***Total***	***Residency* ^a,b^**			**Year of investigation**^***c,d***^				
		***Urban***^***e***^	***Rural***^***f***^		***1991***	***1994***	***1997***	***2000***	
***Health indicators***									
			
Airway resistance [10^-1^•kPa•s/l]	6.30 ± 15.0	6.39 ± 1.49	6.15 ± 1.50	*	5.83 ± 1.53	6.23 ± 1.58	6.96 ± 1.51	6.35 ± 1.27	*
Total lung capacity [% predicted]	105 ± 11	105 ± 10	104 ± 11	-	103 ± 11	105 ± 9	105 ± 9	107 ± 11	*
Birth weight [10•g]	337 ± 59	333 ± 58	348 ± 61	*	334 ± 60	338 ± 56	343 ± 54	337 ± 58	*
Body height [cm]	120.4 ± 5.6	120.0 ± 5.6	121.5 ± 5.4	*	120.6 ± 5.6	120.9 ± 5.4	120.7 ± 5.2	119.6 ± 5.7	*
Overweight, BMI > 19 kg/m^2^	7.5	8.3	4.7	*	5.7	5.1	6.5	11.8	*
Bronchitis, ever diagnosed	45.5	45.0	47.2	-	44.9	52.3	51.2	41.5	-
More than 4 colds in last 12 months	14.7	15.5	11.9	*	15.7	13.2	15.1	13.4	*
Frequent cough	7.8	8.4	5.7	*	8.9	6.8	8.5	6.1	*
Sneeze attacks in the last 12 months	5.8	6.4	3.6	*	4.9	--^g^	6.2	7.1	*
Allergy, ever diagnosed	14.0	14.1	14.0	-	15.8	13.5	12.4	12.1	*
Eczema, ever diagnosed	13.4	13.6	12.6	-	10.7	16.3	15.7	15.5	*
Atopic eczema, day of investigation	7.9	7.0	9.2	-	10.9	9.0	5.5	4.6	*
Specific IgE grass pollen positive	10.8	11.2	10.2	-	9.8	12.2	10.6	10.6	-
Specific IgE house dust mite positive	13.0	13.0	13.1	-	11.7	14.3	10.8	16.2	-
***Living conditions***									
									
Single child	27.2	31.1	13.3	*	28.8	22.1	19.6	29.6	-
Small living space	29.0	33.8	12.1	*	33.8	22.5	20.6	27.4	*
Unfavourable indoor air	16.2	16.3	16.0	-	19.2	23.0	15.8	9.4	*
Damp housing condition	5.3	6.2	2.3	*	5.9	3.9	4.6	5.2	-
Maternal smoking during pregnancy	28.7	33.6	11.4	*	34.8	25.2	19.4	24.3	*
Living near a busy road	50.9	54.6	38.1	*	48.5	57.5	50.6	52.4	*
***Parental education***									
									
Low parental educational level	65.2	66.5	60.6	*	63.4	47.5	67.3	73.5	*

^a ^t-test (continuous variables); ^b ^chi-square test (dichotomous variables); ^c ^t-test for correlation (continuous variables); ^d ^trend test (dichotomous variables); ^e ^Duisburg, Essen, and Cologne; ^f ^Borken; ^g ^not investigated; * p < 0.05

**Table 4:** Distribution of health indicators and living conditions of 6-year-old children in East and West Germany stratified by parental educational level.

	**Mean ± standard deviation^a^/Prevalence [%]^b^**					
	
	***East Germany***			***West Germany***		
	***Parental education***			***Parental education***		
	***Low* ^*c*^**	***High* ^*c*^**		***Low* ^*c*^**	***High* ^*d*^**	
**Health indicators**						
		
Airway resistance [10^-1^•kPa•s/l]	6.71 ± 1.60	6.61 ± 1.62	-	6.41 ± 1.53	6.14 ± 1.45	*
Total lung capacity [% predicted]	103 ± 11	104 ± 10	-	105 ± 11	105 ± 10	-
Birth weight [10•g]	330 ± 53	336 ± 53	*	334 ± 60	342 ± 57	*
Body height [cm]	119.7 ± 5.6	120.6 ± 5.4	*	120.2 ± 5.7	120.6 ± 5.4	*
Overweight, BMI > 19 kg/m^2^	6.6	4.6	*	8.9	4.9	*
Bronchitis, ever diagnosed	43.7	52.8	*	41.9	52.3	*
More than 4 colds in last 12 months	12.0	14.3	*	13.9	16.1	*
Frequent cough	6.6	6.3	-	8.0	7.4	*
Sneeze attacks in the last 12 months	4.5	5.4	*	5.8	5.9	-
Allergy, ever diagnosed	7.8	10.9	*	12.2	17.4	-
Eczema, ever diagnosed	12.9	19.1	*	11.2	17.4	*
Atopic eczema, day of investigation	11.1	12.3	-	7.0	9.3	-
Specific IgE grass pollen positive	9.7	12.8	*	10.1	11.7	-
Specific IgE house dust mite positive	8.5	11.1	*	12.0	14.0	-
**Living conditions**						
						
Single child	38.8	37.6	-	28.4	24.9	
Small living space	51.7	40.8	*	35.4	17.1	*
Unfavourable indoor air	52.3	49.2	*	16.3	16.1	-
Damp housing condition	12.7	8.2	*	6.4	3.3	
Maternal smoking during pregnancy	13.9	4.2	*	36.3	14.5	*
Living near a busy road	62.8	56.7	*	55.0	43.2	*

^a ^t-test (continuous variables); ^b ^chi-square test (dichotomous variables); ^c ^≤ 10 year school; ^d ^> 10 year school; * p < 0.05

**Table 5:** Association of parental educational and living conditions with health indicators of 6-year-old children in East Germany from 1991 to 2000. Results of multiple linear regression and logistic regression analyses.

***Health indicator***		***Parental education***		***Living condition***					
			
		Low parental educational level 1st step *	Low parental educational level 2nd step **	Single child ***	Small living space ***	Unfavourable indoor air ***	Damp housing condition ***	Maternal smoking during pregnancy ***	Living near a busy road ***
	**Sample size**	**Adjusted standardized means difference (95% confidence interval) – bold type if significant (p < 0.05)**							
Airway resistance [kPa•s/l]	1728	1.012 (0.989–1.035)	1.009 (0.985–1.032)	0.994 (0.969–1.019)	1.006 (0.981–1.030)	1.009 (0.980–1.038)	0.995 (0.961–1.030)	1.020 (0.981–1.059)	1.006 (0.981–1.030)
Total lung capacity [% predicted]	1356	0.991 (0.980–1.002)	0.991 (0.980–1.002)	1.005 (0.994–1.017)	1.001 (0.989–1.013)	0.988 (0.975–1.001)	1.007 (0.991–1.023)	0.996 (0.977–1.016)	1.008 (0.996–1.019)
Birth weight [g]	20500	**0.981 (0.977–0.986)**	**0.982 (0.975–0.990)**§a 0.995 (0.986–1.004)§b	**0.985 (0.980–0.990)**	**0.990 (0.985–0.995)**	0.996 (0.991–1.00)	0.995 (0.988–1.003)	**0.953 (0.945–0.961)**	0.998 (0.994–1.003)
Body height [cm]	19087	**0.992 (0.991–0.994)**	**0.992 (0.990–0.995)**	**1.007 (1.005–1.008)**	**0.997 (0.995–0.998)**	0.998 (0.997–1.000)	**0.996 (0.993–0.998)**	**0.990 (0.988–0.992)**	**0.998 (0.997–0.999)**
	**Sample size**	**Adjusted odds ratio (95% confidence interval) – bold type if significant (p < 0.05)**							

Overweight, BMI > 19 kg/m^2^	20547	**1.45 (1.28–1.64)**	**1.44 (1.27–1.63)**	**1.26 (1.10–1.43)**	1.13 (0.99–1.29)	0.89 (0.78–1.01)	0.87 (0.70–1.08)	1.06 (0.87–1.31)	0.96 (0.84–1.08)
Bronchitis, ever diagnosed	19878	**0.71 (0.67–0.75)**	**0.69 (0.65–0.74)**	0.96 (0.91–1.03)	0.98 (0.92–1.04)	1.02 (0.96–1.09)	**1.25 (1.13–1.37)**	1.03 (0.93–1.14)	**1.11 (1.04–1.17)**
More than 4 colds in the last 12 months	20059	**0.83 (0.77–0.91)**	**0.80 (0.73–0.87))**	1.00 (0.91–1.10)	0.99 (0.91–1.08)	**1.13 (1.03–1.23)**	**1.41 (1.25–1.60)**	1.13 (0.98–1.30)	**1.10 (1.00–1.19)**
Frequent cough	20567	1.09 (0.98–1.22)	1.01 (0.90–1.14)	0.98 (0.86–1.11)	0.97 (0.85–1.09)	0.97 (0.86–1.10)	**1.66 (1.42–1.95)**	**1.34 (1.12–1.61)**	**1.29 (1.14–1.45)**
Sneeze attacks in the last 12 months	20123	**0.84 (0.74–0.95)**	**0.78 (0.69–0.90)**	**1.32 (1.15–1.52)**	1.12 (0.97–1.29)	0.92 (0.80–1.06)	**1.52 (1.26–1.83)**	1.12 (0.90–1.39)	**1.29 (1.13–1.48)**
Allergy, ever diagnosed	20071	**0.61 (0.52–0.71)**§a 0.86 (0.70–1.04)§b	**0.60 (0.51–0.70)**§a 0.85 (0.69–1.04)§b	**1.15 (1.04–1.28)**	1.00 (0.91–1.11)	1.07 (0.96–1.18)	1.09 (0.93–1.28)	0.97 (0.82–1.16)	1.10 (1.00–1.22)
Eczema, ever diagnosed	19783	**0.61 (0.57–0.66)**	**0.63 (0.58–0.68)**	1.00 (0.92–1.09)	0.92 (0.85–1.00)	**0.90 (0.83–0.98)**	**1.15 (1.01–1.31)**	**0.80 (0.69–0.93)**	1.03 (0.96–1.12)
Atopic eczema, day of investigation	2021	0.90 (0.68–1.18)	0.92 (0.69–1.21)	1.20 (0.89–1.61)	1.13 (0.84–1.52)	0.74 (0.54–1.01)	1.44 (0.97–2.14)	0.68 (0.39–1.17)	0.92 (0.69–1.22)
Specific IgE grass pollen positive	3204	**0.75 (0.60–0.94)**	0.81 (0.65–1.02)	1.04 (0.82–1.32)	0.91 (0.72–1.16)	**0.70 (0.54–0.90)**	1.03 (0.73–1.43)	0.67 (0.43–1.03)	1.00 (0.79–1.25)
Specific IgE house dust mite positive	3196	**0.74 (0.59–0.94)**	**0.75 (0.59–0.96)**	1.08 (0.84–1.40)	0.99 (0.77–1.28)	0.96 (0.73–1.27)	**1.51 (1.10–2.08)**	**0.62 (0.39–0.99)**	1.04 (0.81–1.32)

* adjusted for gender, year of investigation and urban/rural residency; ** same as '*', but additionally adjusted for the six living conditions; *** same as '*', but additionally adjusted for parental education and the 5 other living conditions; § Interaction between year of investigation and parental education; a 1991; b 2000

**Table 6:** Association of parental educational and living conditions with health indicators of 6-year-old children in West Germany from 1991 to 2000. Results of multiple linear regression and logistic regression analyses.

***Health indicator***		***Parental education***		***Living condition***					
			
		Low parental educational level 1st step *	Low parental educational level 2nd step **	Single child ***	Small living space ***	Unfavourable indoor air ***	Damp housing condition ***	Maternal smoking during pregnancy ***	Living near a busy road ***
	**Sample size**	**Adjusted standardized means difference (95% confidence interval) – bold type if significant (p < 0.05)**							
Airway resistance [kPa•s/l]	983	1.030 (0.999–1.060)	1.015 (0.983–1.047)	0.993 (0.956–1.030)	1.017 (0.980–1.054)	1.022 (0.985–1.059)	0.942 (0.869–1.015)	**1.051 (1.015–1.088)**	1.018 (0.988–1.048)
Total lung capacity [% predicted]	787	1.003 (0.988–1.017)	1.006 (0.991–1.021)	1.017 (0.999–1.035)	0.986 (0.968–1.003)	0.993 (0.976–1.011)	1.026 (0.989–1.063)	0.995 (0.977–1.012)	0.993 (0.978–1.007)
Birth weight [g]	5812	**0.982 (0.972–0.991)**	1.000 (0.990–1.009)	**0.973 (0.962–0.983)**	**0.982 (0.971–0.993)**	0.999 (0.988–1.011)	1.018 (0.999–1.037)	**0.935 (0.925–0.945)**	0.994 (0.985–1.003)
Body height [cm]	5784	0.998 (0.996–1.001)	1.000 (0.998–1.003)	**1.004 (1.001–1.006)**	0.998 (0.995–1.000)	0.999 (0.996–1.003)	1.003 (0.998–1.008)	**0.992 (0.989–0.994)**	1.001 (0.998–1.003)
	**Sample size**	**Adjusted odds ratio (95% confidence interval) – bold type if significant (p < 0.05)**							

Overweight, BMI > 19 kg/m^2^	5783	**1.83 (1.44–2.31)**	**1.67 (1.31–2.14)**	**1.33 (1.05–1.68)**	1.15 (0.90–1.46)	1.12 (0.86–1.47)	1.26 (0.85–1.86)	1.20 (0.96–1.50)	1.03 (0.84–1.26)
Bronchitis, ever diagnosed	5757	**0.67 (0.60–0.74)**	**0.68 (0.60–0.76)**	0.93 (0.82–1.06)	**0.86 (0.75–0.98)**	1.15 (1.00–1.32)	**1.30 (1.03–1.65)**	1.01 (0.89–1.14)	1.01 (0.91–1.12)
More than 4 colds in the last 12 months	5768	**0.83 (0.72–0.97)**	**0.84 (0.72–0.99)**	1.19 (1.00–1.41)	0.95 (0.79–1.14)	0.96 (0.79–1.18)	**1.62 (1.21–2.17)**	**0.83 (0.70–0.99)**	1.11 (0.96–1.29)
Frequent cough	5892	1.12 (0.91–1.37)	0.98 (0.79–1.22)	1.13 (0.89–1.42)	1.16 (0.92–1.46)	0.88 (0.68–1.15)	**2.60 (1.90–3.55)**	1.16 (0.93–1.43)	**1.25 (1.02–1.52)**
Sneeze attacks in the last 12 months	5200	0.94 (0.73–1.20)	0.85 (0.65–1.11)	**1.56 (1.18–2.05)**	1.16 (0.86–1.56)	1.21 (0.88–1.66)	**2.25 (1.52–3.33)**	1.00 (0.76–1.32)	1.11 (0.87–1.42)
Allergy, ever diagnosed	5831	**0.68 (0.59–0.79)**	**0.69 (0.59–0.81)**	1.14 (0.95–1.36)	1.00 (0.83–1.20)	0.97 (0.79–1.19)	1.20 (0.87–1.66)	0.93 (0.78–1.11)	0.95 (0.81–1.10)
Eczema, ever diagnosed	5716	**0.56 (0.48–0.66)**	**0.59 0.50–0.70)**	**0.80 (0.66–0.97)**	0.88 (0.72–1.07)	1.07 (0.87–1.32)	1.10 (0.77–1.57)	0.85 (0.71–1.03)	1.07 (0.92–1.25)
Atopic eczema, day of investigation	2052	0.84 (0.61–1.16)	0.85 (0.61–1.19)	0.92 (0.61–1.40)	0.86 (0.55–1.33)	0.94 (0.62–1.43)	0.71 (0.28–1.82)	0.96 (0.64–1.46)	1.21 (0.87–1.69)
Specific IgE grass pollen positive	1807	0.84 (0.62–1.13)	0.84 (0.61–1.16)	**1.78 (1.25–2.54)**	1.22 (0.82–1.80)	0.84 (0.55–1.27)	0.56 (0.22–1.42)	0.82 (0.55–1.21)	1.09 (0.80–1.48)
Specific IgE house dust mite positive	1781	**0.75 (0.57–1.00)**	**0.72 (0.53–0.97)**	1.22 (0.86–1.73)	1.19 (0.83–1.71)	1.14 (0.79–1.63)	1.18 (0.60–2.30)	0.92 (0.64–1.32)	1.19 (0.89–1.58)

* adjusted for gender, year of investigation and urban/rural residency; ** same as '*', but additionally adjusted for the six living conditions; *** same as '*', but additionally adjusted for parental education and the 5 other living conditions
